# Genomics analysis of *Aphanomyces* spp. identifies a new class of oomycete effector associated with host adaptation

**DOI:** 10.1186/s12915-018-0508-5

**Published:** 2018-04-18

**Authors:** Elodie Gaulin, Michiel J. C. Pel, Laurent Camborde, Hélène San-Clemente, Sarah Courbier, Marie-Alexane Dupouy, Juliette Lengellé, Marine Veyssiere, Aurélie Le Ru, Frédéric Grandjean, Richard Cordaux, Bouziane Moumen, Clément Gilbert, Liliana M. Cano, Jean-Marc Aury, Julie Guy, Patrick Wincker, Olivier Bouchez, Christophe Klopp, Bernard Dumas

**Affiliations:** 1 Laboratoire de Recherche en Sciences Végétales, CNRS UMR5546 Université de Toulouse, Paul Sabatier, 24, chemin de Borde Rouge BP 42617 Auzeville, 31326 Castanet-Tolosan, France; 2 Fédération de Recherche 3450, Plateforme Imagerie, Pôle de Biotechnologie Végétale, 31326 Castanet-Tolosan, France; 3 Laboratoire Ecologie et Biologie des Interactions, UMR CNRS 7267, Université de Poitiers, Poitiers, France; 4 Laboratoire Evolution, Génomes, Comportement, Ecologie CNRS Université Paris-Sud UMR 9191, IRD 247, Gif sur Yvette, France; 5 University of Florida, UF/IFAS, Indian River Research and Education Center IRREC, 2199 South Rock Road, Fort Pierce, FL 34945 USA; 6 Commissariat à l’Energie Atomique (CEA), Institut de Biologie François-Jacob, Genoscope, F-92057 Evry, France; 7 Commissariat à l’Energie Atomique (CEA), Institut de Biologie François-Jacob, Genoscope, CNRS UMR 8030, Université d’Evry, Evry, France; 8 INRA, US 1426, GeT-PlaGe, Genotoul, Castanet-Tolosan, France; 9 INRA, UR875, Plateforme Bioinformatique Genotoul, Castanet-Tolosan, France; 10 Present Address: Plant Ecophysiology, Institute of Environmental Biology, Utrecht University, Utrecht, The Netherlands

**Keywords:** *Aphanomyces*, Oomycete, Effector, Secretome, Host adaptation, SSP

## Abstract

**Background:**

Oomycetes are a group of filamentous eukaryotic microorganisms that have colonized all terrestrial and oceanic ecosystems, and they include prominent plant pathogens. The *Aphanomyces* genus is unique in its ability to infect both plant and animal species, and as such exemplifies oomycete versatility in adapting to different hosts and environments. Dissecting the underpinnings of oomycete diversity provides insights into their specificity and pathogenic mechanisms.

**Results:**

By carrying out genomic analyses of the plant pathogen *A. euteiches* and the crustacean pathogen *A. astaci*, we show that host specialization is correlated with specialized secretomes that are adapted to the deconstruction of the plant cell wall in *A. euteiches* and protein degradation in *A. astaci*. The *A. euteiches* genome is characterized by a large repertoire of small secreted protein (SSP)-encoding genes that are highly induced during plant infection, and are not detected in other oomycetes. Functional analysis revealed an SSP from *A. euteiches* containing a predicted nuclear-localization signal which shuttles to the plant nucleus and increases plant susceptibility to infection.

**Conclusion:**

Collectively, our results show that *Aphanomyces* host adaptation is associated with evolution of specialized secretomes and identify SSPs as a new class of putative oomycete effectors.

**Electronic supplementary material:**

The online version of this article (10.1186/s12915-018-0508-5) contains supplementary material, which is available to authorized users.

## Background

Oomycetes are filamentous eukaryotes that have colonized all terrestrial and oceanic ecosystems [1]. Oomycete evolution started from marine habitats, and their closest cousins are probably free-living phagotrophic protists [1]. The great diversity of lifestyles displayed by oomycetes raised questions about genetic and molecular mechanisms involved in their evolution and rapid adaptation to environmental changes [2, 3]. Oomycete pathogenicity mainly relies on large repertoires of secreted proteins, known as effectors. They show rapid evolution within a given genome as a result of co-evolution with their hosts and are often associated with transfers to unrelated hosts [4, 5]. For instance, protease inhibitors produced by two sister *Phytophthora* species evolved to target plant proteases of their respective unrelated hosts, linking effector specialization and host diversification [6]. *Phytophthora* spp. whole genome studies revealed a bipartite genome architecture that evolved at different rates [5] and where effector genes are associated with transposable elements (TEs) in gene-sparse regions [4]. RxLR and Crinklers (CRNs) are the two main effector families found in these fast-evolving genomic regions of *Phytophthora* spp. [4, 7]. These two large families of effectors consist of modular proteins with a conserved N-terminus host-addressing signal (i.e., a trafficking sequence), while the variable Cter region harbors the effector function [8, 9]. Besides their role in pathogenicity, numerous RxLR proteins are specifically detected by host plants able to produce cognate resistance proteins to trigger resistance [10, 11]. Specific interactions between RxLR effectors and resistance proteins are on the basis of the gene-for-gene concept, a fundamental process of the plant immune system, also known as effector-triggered immunity (ETI) [12]. In that case, rapid evolution of RxLR effectors allows the pathogen to counteract this surveillance system.

Phylogenetic analyses placed the *Aphanomyces* genus in the Saprolegnian lineage, which includes several species pathogenic in plants and aquatic animals such as the salmon pathogen *Saprolegnia parasitica* [13]. In contrast, most of the species in the Peronosporalean lineage are mainly pathogenic on plants, one of the best-studied species being *Phytophthora infestans*, which causes late blight on solanaceous crops such as potato or tomato [8]. The *Aphanomyces* genus has been shown to contain three major lineages, including plant pathogens, aquatic animal pathogens, and saprophytic species [13], making this genus a unique model to understand evolutionary mechanisms involved in adaptation of oomycetes to distantly related hosts and environmental niches. Among the most damaging *Aphanomyces* species is the legume pathogen *Aphanomyces euteiches*, which causes significant damage to various legume crops (peas, alfalfas, faba beans, lentils, etc.) [14]. First reported by Drechsler in 1925 as the causal agent pathogen of root rot in peas in Wisconsin (USA) [15], the pathogen is now recorded in Europe, Australia, New Zealand, and throughout the USA. *Aphanomyces euteiches* is a major problem affecting the field pea in most pea-growing regions [16].

*Aphanomyces euteiches* is composed of distinct subspecific groups based on genotype and host preference (i.e., pea, alfalfa, etc.) [17, 18]. The infection is initiated by oospore germination in close vicinity to a root plant host. The oospores form a germ tube and a long terminal zoosporangium that can release more than 300 primary motile zoospores [19]. The zoospores locate and encyst on the host root within minutes, and the cysts are able to germinate and penetrate the cortical cells within hours. The mycelium then grows intercellularly through the root tissue and forms oospores within a few days of infection [14]. Although the symptoms caused by *A. euteiches* can be difficult to distinguish from symptoms caused by other root-infecting plant pathogens (such as *Pythium* or *Fusarium*), a characteristic colored soft rot of the roots is generally observed [14]. Under optimal field conditions, legume infection by *A. euteiches* can result in symptoms within 10 days, and oospores can be detected between 7 and 14 days [14]. The oospores of *A. euteiches* are long-lived and can remain dormant in soil and organic debris for many years [14], making legume cultivation inefficient. Effective chemical controls for *Aphanomyces* root rot of legumes are not available, and the development of tolerant cultivars appears to be the more effective management technique available to farmers. Partial resistance in pea or the legume model *Medicago truncatula* to *A. euteiches* is mediated by several quantitative trait loci (QTLs). Recent whole genome sequencing data in conjunction with genome-wide association studies (GWASs) on *M. truncatula* have allowed the identification of promising candidate genes [20, 21] to manage the parasite.

*Aphanomyces astaci* is an obligate parasite of freshwater decapods, particularly crayfish (but crabs could act as potential vectors as well [22, 23]). It presumably originates from North America, where infected native crayfish do not show disease symptoms. Five distinct *A. astaci* genotype groups (from A to E) are known in Europe and were isolated from infected European crayfish specimens. *A. astaci* reproduces asexually through the formation of short-lived bi-flagellated zoospores that spread in aquatic ecosystems [23]. After the encystment of the zoospore on the host cuticle and its germination, the growing hypha penetrates into the cuticle. In resistant crayfish, the hyphal growth is stopped by encapsulation and melanization, resulting from the host immune response. In susceptible crayfish, hyphae penetrate deeper into tissues and organs, generally killing the host [24]. The time required for development varies depending on the *A. astaci* strain and water temperature [25]. Like plant pathogens, *A. astaci* presumably secretes a battery of virulence proteins to promote infection and facilitate host adaptation. Indeed, infection experiments have shown that *A. astaci* strains from group E had a high level of virulence, comparable to that of group B [26]. *A. astaci* has been nominated among the “100 of the World’s Worst Invasive Alien Species” in the Global Invasive Species Database [27].

In contrast to species from the Peronosporalean lineage, little is known regarding the molecular mechanisms that govern host adaptation and environmental niche colonization for *Aphanomyces* species. A previous analysis of a collection of expressed sequence tags (ESTs) from the legume pathogen *A. euteiches* revealed the uniqueness of its effector repertoire, which is composed of numerous CRN effector genes without any RxLR effectors [28–30]. This observation led to the suggestion that CRNs are ancestral in the oomycete lineage and specific to phytopathogenic species [7, 9]. Indeed, CRN coding genes have been detected in all plant pathogenic oomycetes sequenced to date, but never in animal pathogenic species such as *S. parasitica* [31]. This has also raised the possibility that, besides the RxLR family, CRNs and maybe other oomycete effectors may have important roles in triggering host susceptibility.

Here we report on the whole genome sequencing and annotation of a pea-infecting strain of *A. euteiches*. To characterize genetic factors involved in plant adaptation by comparative genomics, we generated a first genomic draft of the crustacean pathogen *A. astaci*. In combination with RNA-seq data, we showed that *Aphanomyces* adaptation to plant or animal hosts is correlated with the expression of highly specialized secretomes. Moreover, our study led to the identification of a set of small secreted proteins (SSPs) which can be considered as a new class of oomycete effectors.

## Results

### *Aphanomyces* spp. genome sequencing and phylogeny relationship

The genome of the *A. euteiches* ATCC201684 strain was sequenced by combining 454 and Illumina sequencing technologies providing a high-quality reference assembly of 57 Mb (Table 1). The estimated genome size of *A. euteiches* is consistent with the size range of most previously sequenced oomycete genomes except *P. infestans* (240 Mb) [32] and close to the assembled *S. parasitica* fish pathogen genome (63 Mb) [31]. The GC content (~ 47%) is one of the lowest detected in oomycetes and in close agreement with the *Albugo laibachii* [33] and *Plasmopara viticola* genomes [34]. The benchmarking universal single-copy orthologs (BUSCO) method [35] was used to estimate the degree of completeness of the assembled gene space. Most of the gene space was covered as 83.1% complete, and a 3.8% partial model of conserved fungal genes was identified within the genome of *A. euteiches*. The number of genes (19,548) is more important in *A. euteiches* than in other sequenced oomycetes, while the gene length mean (~ 1.5 kb) is similar to that of various oomycetes [33, 34, 36]. The *A. euteiches* genome is thus highly compact with the second-highest gene density (one gene per 2.9 kb) reported so far for oomycetes and similar to the *S. parasitica* genome (one gene per 2.6 kb). The completeness of the *A. euteiches* sequenced genome and prediction of open reading frames were validated by the analyses of expressed sequences. In total, 87% of ESTs previously generated from *A. euteiches* mycelium (MYC) and mycelium grown in contact with *M. truncatula* roots (INT) [28] mapped to the assembly. In addition, ~ 90% of assembled Illumina reads generated during this study by using MYC and INT samples, mapped to the *A. euteiches* genome assembly. The completeness of the assembly was further evaluated with the BUSCO method [35] using the Alveolata-Stramenopiles dataset. The same analysis was performed with eight sequenced oomycetes (Additional file 1: ST1a, b). The *A. euteiches* assembly contained > 94% of the Stramenopiles dataset and appears as the better one among the sequenced oomycetes. All the data were included in an updated version of the AphanoDB database dedicated to “omics” studies on the *Aphanomyces* genus [37]. AphanoDB v2.0 provides new tools as a genome browser, gene annotation facilities, and Basic Local Alignment Search Tool (BLAST) tools.

**Table 1:** Main features of *A. euteiches, A. astaci*, and *A. stellatus* genomes

	*A. euteiches* ^*a*^	*A. astaci* ^*b*^	*A. stellatus* ^*b*^
Estimated genome size (Mb)	61	94	71.6
Total contig length (Mb)	56.9	45.3	62.1
GC content (%)	47.69	48.51	52.55
Protein-coding genes	19,548	16,479	25,573
Average exons per gene	3.7	2.7	3.2
Mean gene size (kb)	1.503	1.132	1.467
N50 (bp)	275,164	3659	34,018
Gene density (number of genes per Mb)	343	364	412

^a^Combination of 454 and Illumina sequencing technologies

^b^Illumina sequencing technology

Genome sequences and assemblies of the crayfish pathogen *A. astaci* and the opportunistic pathogen *A. stellatus* were generated, giving an estimated size of 45 Mb and 62 Mb (Table 1) with 16,479 and 25,573 predicted genes, respectively. As expected, genome assemblies of *A. astaci* and *A. stellatus* compiled only from short reads generated by Illumina are more fragmented and must be considered as drafts.

**Fig. 1:**
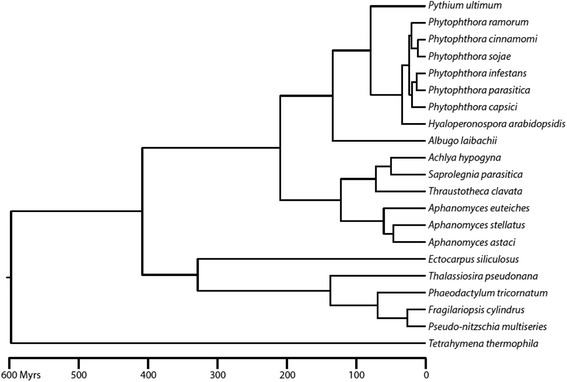
Phylogenetic position and divergence times of *Aphanomyces* spp. The phylogeny was inferred by a maximum likelihood analysis of 40 concatenated genes [38]. All nodes of the tree are supported by bootstrap values of 100. Branch lengths are proportional to absolute time. Divergence times were inferred with the same calibration points and parameters as those implemented in Matari and Blair [38]

These data were used to perform a phylogenetic analysis with a gene set recently used to estimate timescale evolution of oomycetes [38] (see Methods for further details). *Aphanomyces* species form a group which diverged from other Saprolegniales (*Saprolegnia parasitica, Achlya hypogena*, *Thraustotheca clavata*) more than 100 Mya (Fig. 1). This analysis also shows that divergence times are very deep within the *Aphanomyces* genus, with the three *Aphanomyces* species diverging from each other more than 50 Mya.

### Transposable Elements (TE)

**Fig. 2:**
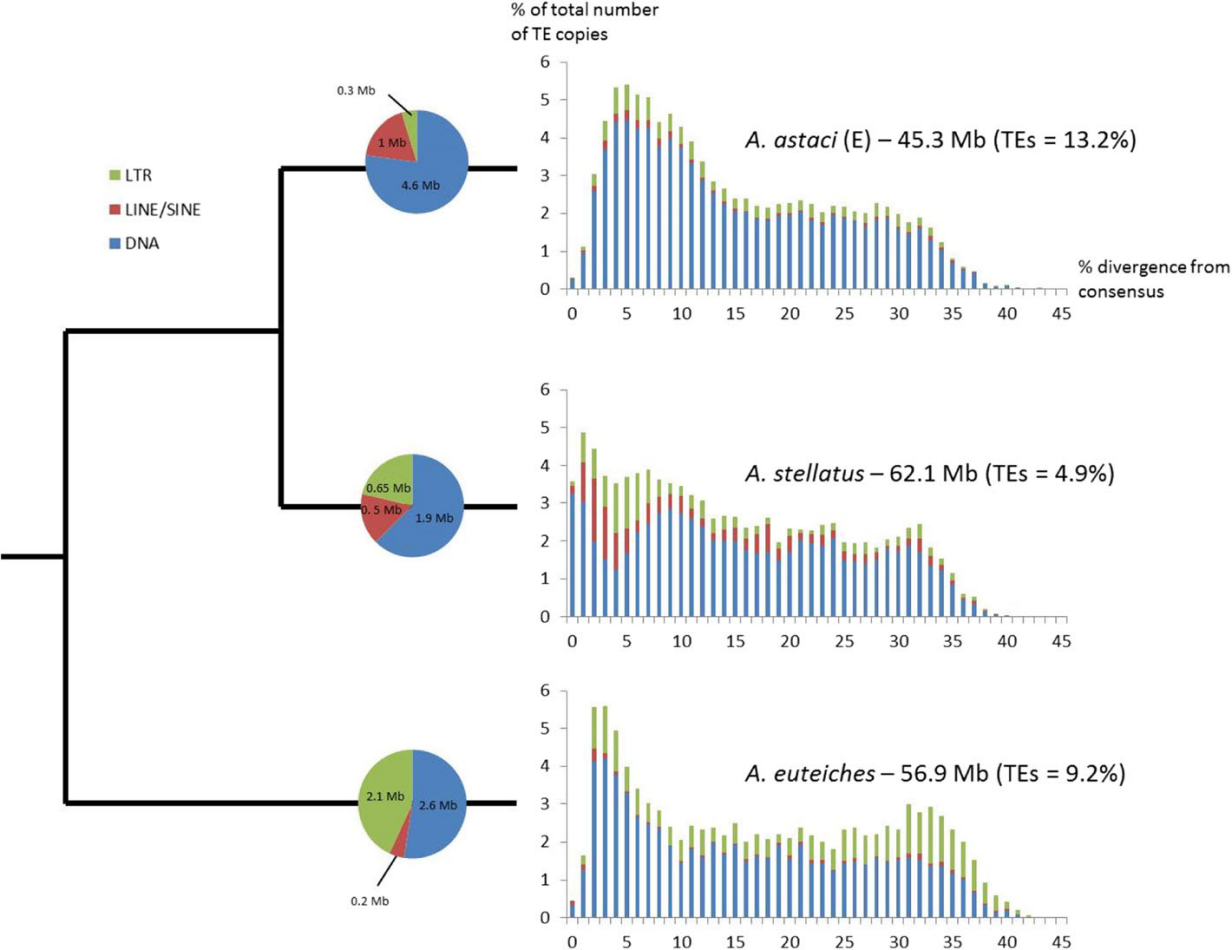
Evolutionary dynamics of transposable elements in *Aphanomyces* spp. genomes. Histograms on the *right side* of the figure correspond to the frequency distribution of percent divergence between all TE copies and their cognate consensus sequence for Class I long-terminal repeat retrotransposons (*LTR*) and long and short interspersed elements (*LINE/SINE*), as well as Class II DNA transposons (*DNA*). Note that the low amount of copies showing percent identity > 40% to their consensus can be due either to the absence of such copies or to the fact that we were unable to detect them. Venn diagrams illustrate the number of bases (in megabases) occupied by each category of TE in the three *Aphanomyces* genomes

TEs are known to play a prevalent role in the evolution of eukaryotic pathogens [2, 3]. A de novo characterization of TEs was thus performed on all *Aphanomyces* genomes. The aim of the TE analysis was to uncover and annotate all TE copies in all three *Aphanomyces g*enomes to facilitate future studies that will investigate the origin, evolution, and genomic impact of these elements in more detail. The fraction of *Aphanomyces* genomes occupied by TEs (5–13%; Additional file 1: ST1d) is lower than that for all other sequenced oomycetes (17–74%) in which TEs have been mined, such as *Phytophthora* species [32, 39], the downy mildew *Plasmopara viticola* [35], and the white rust *Albugo laibachii* [34], with the exception of *Pythium ultimum* (5%) [40]. It is noteworthy that in oomycetes genome size is strongly correlated with TE content (*R*^2^ = 0.85), supporting the hypothesis that TEs are a major determinant of genome size in these taxa [32]. In all three *Aphanomyces* species, the most abundant TEs are DNA transposons of the Tc1-mariner superfamily (up to 2.5% of the genome in *A. astaci*). Interestingly, none of the TEs that we identified are shared between all three *Aphanomyces* species, and very few TEs are shared between two species: two are shared between *A. astaci* and *A. euteiches* (74 and 83% identity over 741 and 170 bp respectively), two are shared between *A. stellatus* and *A. euteiches* (84 and 86% identity over 334 and 1262 bp respectively), and four are shared between *A. astaci* and *A. stellatus* (between 78% identity over 1820 bp and 84% identity over 195 bp) (Fig. 2). Furthermore, BLASTN searches using all TEs identified in *Aphanomyces* spp. as queries against all Repbase TEs did not reveal any significant hit. Together, these results suggest that *Aphanomyces* species TE families are characterized by a high rate of turnover.

### Comparative analysis of *A. euteiches* and *A. astaci* proteomes

**Fig. 3:**
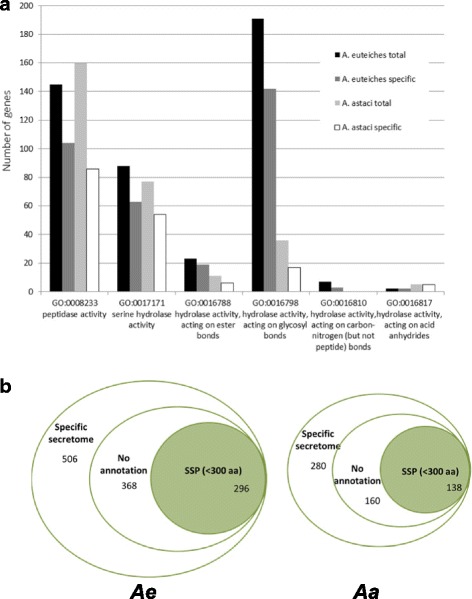
Functional annotation of the plant pathogen *A. euteiches* and the crustacean pathogen *A. astaci.* **a** Number of GO terms related to “hydrolase activity” category (Molecular Function) detected in *A. euteiches* and *A. astaci* secretomes. Genes detected in *A. euteiches* and not in *A. astaci* are described as *A. euteiches specific* and vice versa. **b** OrthoMCL analysis of *A. euteiches* and *A. astaci* proteomes against nine deeply sequenced oomycetes (*S. parasitica, P. infestans, P. ramorum, P. parasitica, P. sojae, Py ultimum, Py irregular, A. laibachii, Hyaloperonospora parasitica*). Graphics are illustrated in terms of number of genes and the specific secretome content of *A. euteiches* (*Ae*, *left*) and *A. astaci* (*Aa*, *right*). Note the presence of small secreted protein (*SSP*) (less than 300 amino acids in size, without functional annotation) in both *Aphanomyces* species

To identify conserved and specific features of *Aphanomyces* proteomes, an OrthoMCL analysis [41] was performed using *A. euteiches* and *A. astaci* proteomes and nine deep-sequenced oomycete proteomes (*S. parasitica*, *P. infestans*, *P. sojae*, *P. parasitica*, *P. ramorum*, *Hyaloperonospora arabidopsidis*, *A. laibachii*, *Py. ultimum*, and *Py. irregulare*) (Additional file 1: ST1e). A total of 2296 orthologous groups (34,404 genes) were detected in the 11 genomes defining a “core proteome” set (Additional file 1: ST1f). In this set 104 groups corresponding to 1528 genes defined the “core secretome” (Additional file 1: ST1f). A focus on the *A. euteiches* secretome (1240 genes, ~ 6% of the proteome, Additional file 1: ST1g) shows that 70% of the sequences harboring a Gene Ontology (GO) term are related to the “hydrolase activity” category (GO:0016787) (Additional file 1: ST1h and Fig. 3a). This category is enriched in enzymes with glycosyl hydrolase or peptidase functions and other typical oomycete secreted proteins, such as protease inhibitors. The secretome of *A. astaci* is predicted to contain 744 genes (< 5% of the proteome). While this set is probably not complete, 323 sequences harbor a GO annotation and more than 65% (217 sequences) fall into the “hydrolase activity” category with a predominance of “peptidase GO:0008233” function (160 sequences, 73%). A closer examination of both secretomes showed that numerous genes detected in *A. euteiches* are not reported in *A. astaci* (Fig. 3a). In addition, about 72% of the *A. euteiches*-specific secretome (i.e., sequences not present in other oomycetes including *A. astaci*, 506 genes) did not display a putative functional domain (368 genes), and 80% encoded proteins below 300 amino residues in size (296/368, Additional file 1: ST1i, Fig. 3b). The same observation was made for the *A. astaci*-specific secretome, where 160 specific genes did not harbor a functional annotation and 86% (138/160, Additional file 1: ST1i) coded for small proteins (Fig. 3b). Interestingly, 59% of these small proteins were predicted to be putative effectors from *A. euteiches* or *A. astaci* using EffectorP (Additional file 1: ST1i), a software based on properties shared by fungal effectors [42]. This suggests that *Aphanomyces* species presented a set of SSPs similar to fungal effectors.

### Carbohydrate-Active enZymes (CAZy) and carbohydrate-binding modules (CBMs)

**Fig. 4:**
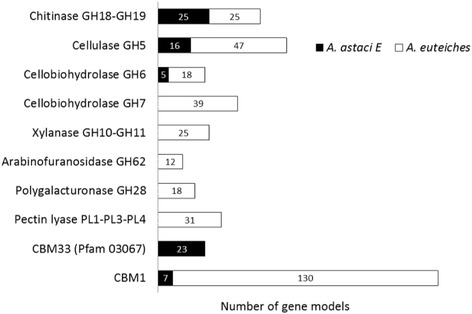
Most representative CAZyme families predicted in *A. euteiches* and *A. astaci* genomes. Repertoires of CAZymes predicted in *A. euteiches* (*white squares*) and *A. astaci* (*black squares*) are correlated to their respective host polysaccharides

Since comparative analyses suggested that the repertoires of glycosyl hydrolases are divergent between *A. euteiches* and *A. astaci*, a global prediction of CAZy domains (glycosyl hydrolases (GH), carbohydrate esterases (CE), polysaccharide lyases (PL), and carbohydrate-binding modules (CBM)) was performed. This analysis revealed that the *A. euteiches* genome is enriched in gene coding for proteins with CAZy domains compared to the *A. astaci* genome (Fig. 4, Additional file 1: ST1j). For example, more than 300 genes coding for proteins with at least one predicted GH domain were detected in the *A. euteiches* genome, a repertoire size similar to those predicted in oomycete genomes such as *Phytophthora* species [43], whereas only 109 genes were found in the *A. astaci* genome.

A closer examination of this repertoire revealed that several enzyme families targeting plant-specific polysaccharides (pectins, hemicellulases) are expanded in *A. euteiches* and absent in *A. astaci.* Clearly, the *A. astaci* genome lacks genes coding for hemicellulases (e.g., GH, 10, 11, CE4 families) and pectinases (e.g., GH28, PL1, PL3, and PL4), which are largely represented in the *A. euteiches* genome (Fig. 4, Additional file 1: ST1j). Based on the CAZy database, eight GH families correspond to eukaryotic cellulases. The GH5, GH6, and GH7 families, which encode endocellulases and cellobiohydrolases, are highly represented in *A. euteiches* as compared to other oomycetes. Since oomycete cell walls contain cellulose, these enzymes can play a role in oomycete cell wall remodeling. Interestingly, while GH5 and GH6 cellulases are present in *A. astaci* and *A. euteiches*, a large family of GH7 was found only in *A. euteiches* (39 GH7-encoding gene), suggesting that this class of enzymes could play a role in plant pathogenesis (Fig. 4).

In phytopathogenic fungi it is suggested that α-l-arabinofuranosidases such as GH62 and GH54 are involved in plant penetration and pathogenesis [44, 45]. While GH62 are absent in oomycetes belonging to the Peronosporalean lineage analyzed so far, 12 putative GH62-coding sequences are detected in *A. euteiches*, while GH54 are missing (Fig. 4, Additional file 1: ST1j). Among GH62, seven are predicted to be secreted. It has also been suggested that α-l-arabinofuranosidases, by degrading arabinofuranose side chains, improve the accessibility of the xylan backbone of hemicellulose to xylanases as GH10 and GH11 [46], which are present in *A. euteiches* (22 and 3 sequences respectively). Thus, the presence of genes coding for these enzymes suggests that *A. euteiches* can express a complete repertoire of hemicellulose-degrading enzymes, distinct from those produced by other oomycetes.

The availability of CAZyme repertoires from distantly related oomycetes offers the opportunity to investigate their origin. Since most of these genes are closely related to fungal genes, it has been suggested that some of them were acquired by oomycetes by horizontal gene transfer (HGT) events from true fungi [47]. In certain cases, this hypothesis was sustained by phylogenetic analyses. This is the case for a pectate lyase gene from *P. infestans* (EEY64154) [48] for which seven orthologous genes, corresponding to the PL1 family, were detected in the *A. euteiches* genome but not in *A. astaci* (Additional file 1: ST1j). Phylogenetic analysis using these sequences (Additional file 1: ST1k, Additional file 2: Figure S1) supports the hypothesis that these genes were acquired early in the evolution of oomycetes, before the Saprolegniale-Peronosporale divergence, and probably lost during the specialization of *Aphanomyces* species to animal hosts.

Numerous CAZymes are associated with CBMs to facilitate enzyme activity [49]. In *A. euteiches*, many CAZymes are associated with CBMs, and 130 CBM1 (cellulose-binding modules) predicted proteins are detected in this species, but only 7 genes in *A. astaci* (Additional file 1: ST1j, Fig. 4). In contrast, *A. astaci* harbors a set of genes encoding proteins with a putative chitin-binding domain (PF03067, IPR004302, CBM33, now reclassified as the AA10 domain in CAZy) that are not detected in *A. euteiches* (Additional file 1: ST1j, Fig. 4). Interestingly, > 80% (19/23) of these domains are associated with a predicted signal peptide. These data suggested that CBM33 from *A. astaci* might play a role in the interaction with the crustacean shell, as reported for the PlCBP49 CBM33-containing virulence factor of the honey bee pathogen *Paenibacillus larvae* that degrades chitin [50].

### *Aphanomyces* spp. cytoplasmic effectors (RxLR and CRN oomycete effectors)

An important discovery regarding oomycete genome sequencing projects resides in the identification of predicted secreted proteins that could be delivered into host cells to aid pathogenicity. Two types of cytoplasmic effectors dominate the oomycete secretome: the RxLR effectors and the Crinklers (CRNs). These effector proteins are characterized by an amino acid signature located at the N-terminus of the sequence. We thus investigated the presence of these host-targeting signals in *A. euteiches* and *A. astaci* species. The genomes of both *Aphanomyces* were searched for RxLRs and CRNs as previously reported [17, 40]. This approach did not allow the identification of RxLR effectors on both *Aphanomyces* genomes, as previously suggested upon analysis of EST libraries from the same strain of *A. euteiches* [17]. This analysis sustains the view that RxLR effectors seem to be absent in the Saprolegniale lineage [20]. For predicting CRNs, a combination of automatic searches (hidden Markov model (HMM), regular expression) and manual curation methods was used, and 160 and 31 CRNs and CRN-like genes were detected in the *A. euteiches* and *A. astaci* genomes respectively (Additional file 1: ST1k). For *A. euteiches*, this number is similar to the one reported in phytopathogenic *Phytophthora* species (> 80 *P. capsici*; > 200 *P. sojae*; > 400 *P. infestans* [32, 51]).

Consistent with previous data on oomycete genomes [7, 52, 53], fewer than 25% of *A. euteiches* CRNs and none in *A. astaci* were predicted to be secreted by means of SignalP analysis. By contrast, around 60% of AeCRNs and AeCRN-like genes harbor a predicted nuclear-localization signal (NLS). Among them 16 have both a predicted signal peptide and an NLS. A majority of AeCRNs (> 60%) harbor a LYLAK motif at the N-terminal rather than the canonical *Phytophthora* LxLFLAK motif, as previously reported upon complementary DNA (cDNA) annotation of *A. euteiches* [28, 29, 54]. We noticed that the N-terminal trafficking signal is less conserved in *A. astaci* as compared to CRNs from *A. euteiches*, and only five sequences harbor a putative header signal in combination with a C-terminal domain (Additional file 1: ST1k). The headers of CRNs are followed by a more diverse C-terminal domain that confers effector activity [32]. Based on sequence similarity, 36 domains were initially defined for the CRN repertoire of *P. infestans*, and new C-termini domains have been characterized upon the CRN repertoire annotation of various oomycete species [53, 55]. HMM searches and manual assignment on AeCRNs and AeCRN-like sets showed that the necrotic DXZ, D5, and D2 kinase domains are among the most widespread C-termini domains in *A. euteiches* (Additional file 1: ST1k). The DC domain, reported as a putative DNA-binding helix-hairpin-helix (HhH) motif in PsCRN108 from *P. sojae* [56], is also largely represented in *A. euteiches* but not in *A. astaci.* The DXZ and DN17 domains frequently detected in phytopathogenic oomycetes and the chytrid *Batrachochytrium dendobraditis* [32, 55, 57] are well represented in *A. astaci*.

### Dynamic changes of *A. euteiches* transcriptome during infection of *M. truncatula* roots

To get an insight into the expression and regulation of *A. euteiches* genes during different life stages, RNA-seq analyses were performed on RNAs isolated from *A. euteiches* zoospores and mycelium grown in liquid culture as well as in infected *M. truncatula* roots harvested 1, 3, and 9 days post inoculation (dpi). For the zoospores and the in vitro mycelium libraries, around 50 M reads were obtained, of which 70–77% were mapped in pairs to gene-coding regions (Additional file 3: ST2a). For *M. truncatula*-infected roots, 46—71 M reads were obtained, of which most could not be mapped to the *A. euteiches* genome, since a high amount of *M. truncatula* material was present in the samples. In addition, since less than 1% of the reads of 1 dpi could be mapped to the *A. euteiches* genome, this time point was excluded from the analysis (data not shown). For time points 3 and 9 dpi, 6–13% of the reads could be mapped in pairs to gene-coding regions (Additional file 3: ST2a). After filtering for lowly expressed genes and data normalization, a multi-dimensional scaling plot revealed a clear separation between expression patterns of the different life stages of *A. euteiches*, while the biological repeats of each life stage (mycelium, zoospore, 3 dpi, and 9 dpi) clustered together (Additional file 4: Figure S2). This plot suggested that different gene subsets are expressed during *M. truncatula* infection.

For the 16,786 (85.8%) genes that were expressed, differential expression between the different life stages was determined. Around 36–37% of the genes were differentially expressed between zoospores and the other life stages, while only 10–11% of the genes showed differential expression between the in vitro grown *A. euteiches* mycelium and the infected root material (Additional file 3: ST2b). This could reflect a transcriptome remodeling during host infection. To further investigate in what processes the differentially expressed genes are involved, a Gene Ontology (GO) enrichment analysis was performed. During infection, genes that are upregulated between 3 and 9 dpi are mainly involved in “carbohydrate metabolic (GO:0005975)” and “proteolysis (GO:0006508)” processes, while downregulated genes belonged mainly to transport processes (GO:0055085, GO:0030001). The opposite situation is detected when comparing zoospores with in vitro grown mycelium (Additional file 3: ST2c–e), suggesting important physiological differences between zoospores and mycelium life stages of *A. euteiches* as reported for *Phytophthora infestans* [58].

**Fig. 5:**
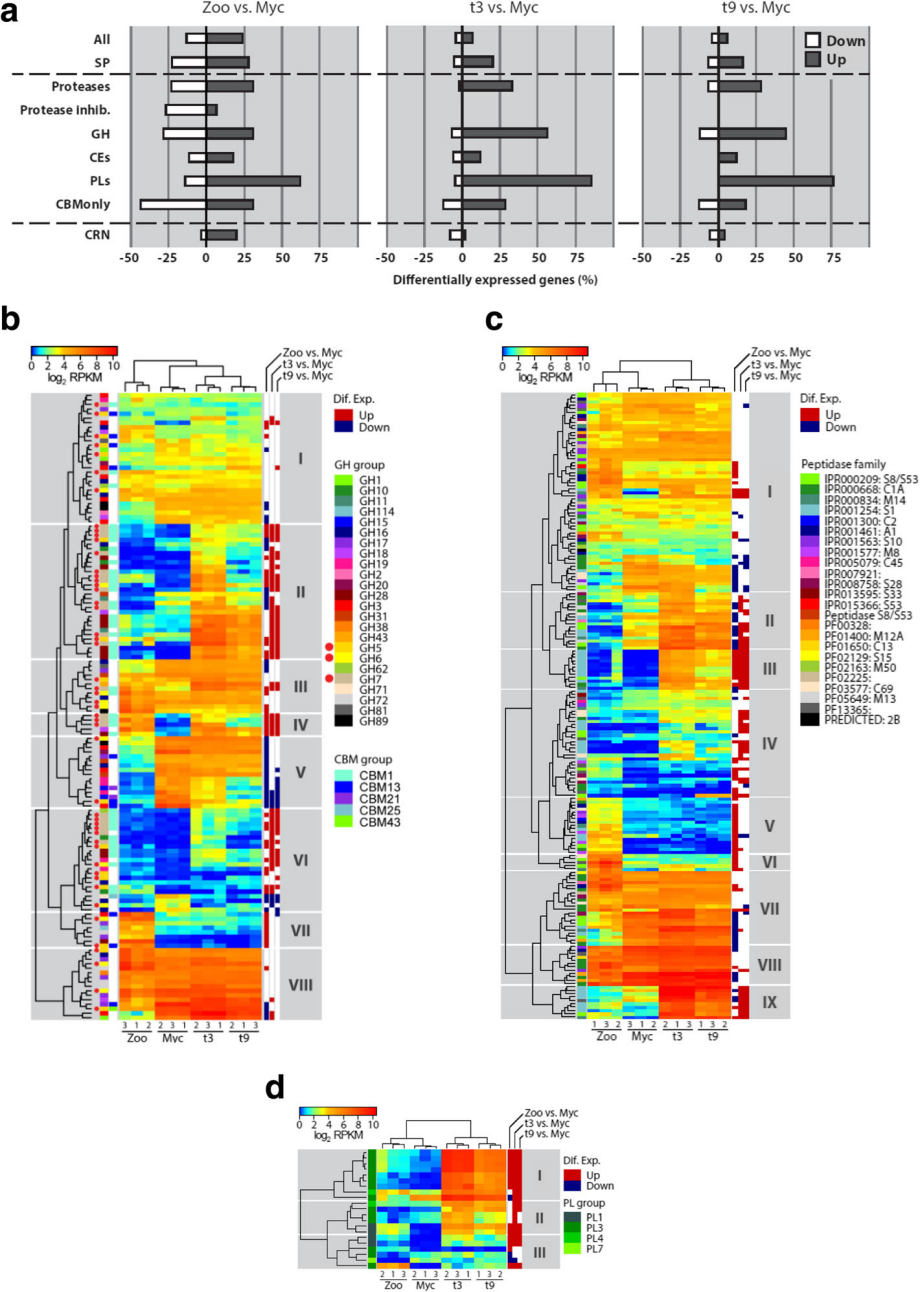
Gene expression of *A. euteiches* at different life stages and during *M. truncatula* infection. **a** The percentage of up- and downregulated genes in zoospores, infected roots 3 days post infection (dpi), and infected roots 9 dpi compared to mycelium grown in vitro for all expressed genes and different subsets of expressed genes. (*All* all genes, *SP* genes with a predicted signal peptide, *Proteases* secreted proteases, *Protease inhib*. secreted protease inhibitors, *GH* secreted glycoside hydrolases, *CEs* secreted carbohydrate esterases, *PLs* secreted polysaccharide lyases, *CBMonly* secreted carbohydrate-binding module containing genes without a catalytic domain, *CRN*, Crinklers and Crinkler-like). **b–d** Heatmaps of the log2 reads per kilobase per million (*RPKM*) values of the glycoside hydrolases (**b**), proteases (**c**), and polysaccharide lyases (**d**) with a predicted signal peptide. *Colors on the left* of the heatmaps indicate the subgroup that the GH, protease, or PL belongs to and, if present, the group of carbohydrate-binding module present in the gene. *Colors on the right* indicate if the genes are significantly up- (*red*) or down- (*blue*) regulated in zoospores, infected roots 3 dpi, and infected roots 9 dpi compared to expression in mycelium grown in vitro. The *red dots* indicate GH groups with predicted cellulose activity

We then investigated the expression pattern of the *A. euteiches* secretome with a focus on CAZymes, proteases and protease inhibitors, CBMs, and CRNs/CRN-like effectors (Fig. 5a). When zoospores and mycelium stages are compared, most groups of genes encoding secreted proteins show a similar percentage of differential expression with the exception of the “polysaccharide lyase (PL)” category, which displays the highest percentage of differentially expressed genes. In contrast, during infection (3 and 9 dpi) around three times as much of the genes coding for secreted proteins are upregulated, with proteases, glycoside hydrolases (GH), and PL (86% and 76%) showing the highest percentages of upregulated genes (Fig. 5a). Less than 2% of CRN genes are upregulated at 3 and 9 dpi, while 13% are upregulated in zoospores as compared to in vitro grown mycelium, suggesting that a subset of AeCRN is present at the early stage of *Medicago* infection (Fig. 5a).

Heatmaps to visualize expression level of genes rather than differential expression show that, for GH, several groups can be identified (Fig. 5b, Additional file 3: ST2f) with groups II, IV, and VI containing genes that are upregulated during infection compared to mycelium. These groups contain a large number of genes coding for proteins with predicted cellulase (GH5, GH6, and GH7) and polygalacturonase (GH28) activity. They also include numerous hemicellulase genes (GH10, GH11) and one secreted arabinofuranosidase (GH62). Most of the polysaccharide lyases (PL) are also strongly induced during infection (Fig. 5d, Additional file 3: ST2g). Secreted PL1 are detected in group II or III, where gene expression is less abundant in mycelium as compared to zoospore and infection stages. This pattern is similar to the one observed in *Phytophthora capsici,* where the PL1 family in combination with PL16 and PL20 (absent in the *Ae* genome) account for nearly all of the contribution of the 22 PL genes to *Phytophthora* virulence [59]. Most of the carbohydrate esterases (CE) showed a consistent low to medium expression in the different life stages (Additional file 5: Figure S3A, Additional file 3: ST2i). For the CBM-containing proteins without a predicted catalytic site mostly composed of CBM1 domains (cellulose-binding), several groups of genes can be identified with group II being strongly induced and expressed during infection while groups III and V are specifically upregulated or downregulated respectively in the zoospore stage (Additional file 5: Figure S3B, Additional file 3: ST2j). This expression pattern suggested that *A. euteiches*-secreted CBM1 domains may contribute either to *A. euteiches* virulence or microorganism development, as reported for the CBM1-containing protein CBEL from *P. parastica* that mediates adhesion to cellulosic substrates and contributes to *Phytophthora* cell wall architecture [60, 61].

For the secreted peptidases, several groups can also be identified (Fig. 5b, Additional file 3: ST2h). Many peptidases show constitutive expression during all life stages (groups I, VII, and VIII), while some are induced in zoospores (groups V and VI) or during *M. truncatula* root infection (groups II, III, IV, and IX). These last groups contain a high number of serine proteases (S1 family) that could be considered as candidates for pathogenicity factors. Indeed, serine proteases have been characterized as the major extracellular proteolytic enzymes secreted by *Phytophthora* spp. and indispensable for necrosis [62]. Notably, none of the secreted protease inhibitors is differentially expressed during infection. However, most of the secreted protease inhibitors are highly expressed during all life stages of *Ae* (Additional file 5: Fig. 3C, Additional file 3: ST2k).

Together, these analyses indicate that expression of several families of genes coding secreted proteins are induced in zoospores and during infection of *M. truncatula* roots compared to saprophytically grown mycelium. This supports an important role in the pathogenesis of cell wall-degrading enzymes specifically found in *A. euteiches* and not in *A. astaci* targeting plant cell wall polysaccharides (pectins, hemicellulose).

### Identification and functional characterization of small secreted proteins in *A. euteiches* (AeSSPs)

As reported above, 40% of *A. euteiches*-specific genes that encoded putative secreted proteins did not display a functional annotation and are below 300 amino acid residues in size (< 300 genes, Fig. 3b, Additional file 1: ST1i). Consequently, putative AeSSPs represented 24% of the secretome. Around 5% are organized in clusters (≥ 3 adjacent SSP genes, Additional file 6: ST3a). The clusters are distributed all over the genome and comprise 3 to 9 genes each. The clusters mainly contain AeSSP genes from the same OrthoMCL group, indicating that they might have arisen by duplication.

**Fig. 6:**
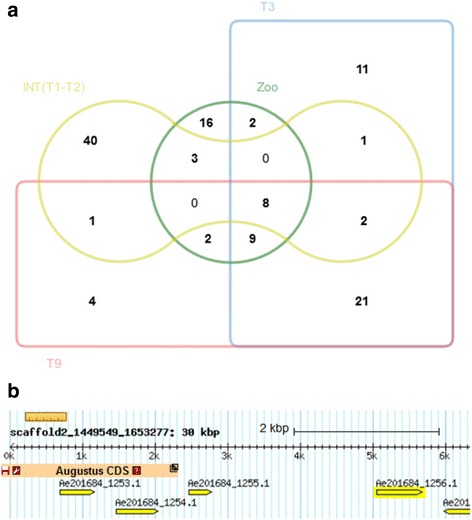
Small secreted protein from *A. euteiches* (AeSSP). **a** Edwards diagram depicting number of AeSSP genes that are upregulated (FC > 2) either during in vitro growth as zoospores (*Zoo*) or during host infection (*T3*, *T9*, *INT(T1-T2)*), compared to mycelium grown condition. **b** Screen shot of AphanoDB v2.0 illustrating an AeSSP cluster from *A. euteiches* (*AeSSP_1253 to AeSSP_1256*) of 296 genes. All genes in the cluster are specific to *A. euteiches,* strain ATCC201684, contain a predicted signal peptide, are less than 300 amino acids in size, and do not harbor any functional domain. Note that AeSSP1254 and AeSSP1256 harbor a predicted nuclear localization signal (NLS)

To ascertain the expression of AeSSPs during host infection, we checked the expression data presented above (e.g., mycelium, zoospore, interaction 3 dpi (T3), interaction 9 dpi (T9)). Since we could not exploit RNA-seq data generated at 1 day post infection, we included data from our previous set of cDNA generated from mycelium (MYC library) and *A. euteiches* grown in contact with roots during 1 or 2 days (INT T1 + T2, [28] (Additional file 6: ST3a). As shown in Fig. 6a, a large set of AeSSPs are upregulated in zoospores, in mycelium in contact with plant roots, or during infection. In total, 120 AeSSPs have been found to be induced in at least one condition.

**Fig. 7:**
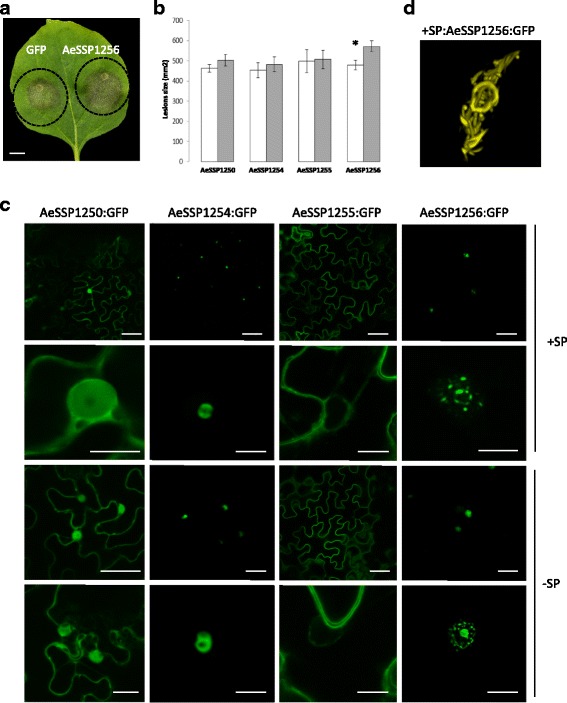
AeSSPs are nuclear localized and enhance susceptibility to oomycete infection. **a** *Agrobacterium tumefaciens* was used to transiently express GFP on one half of a 3- week-old leaf of *N. benthamiana*, while AeSSP candidate fused to GFP was expressed on the other half of the leaf. One day after treatment, the infiltrated area was inoculated with *Phytophthora capsici* zoospore. Photograph (3 dpi) illustrated symptoms observed upon AeSSP1256 expression vs GFP. *Black line* indicates the agroinfiltrated area. Scale bar = 1 cm. **b** The average lesion sizes (mm^2^) were quantified 3 days after *P. capsici* inoculation. Results presented the means +/− standard error of the mean (SEM) of independent experiments. *Asterisk* indicates significant differences (Student’s *t* test, *p* < 0,05). **c** *A. tumefaciens* was used to transiently express either a full-length version of GFP-tagged AeSSPs (*+SP, top panels*) or a matured form (–*SP, lower panels*). Photographs are taken 24 hours post inoculation (hpi). Scale bars = 5 μm. **d** Closer view of the subcellular localization of the full-length version of AeSSP1256 after transient expression in *Nicotiana* leaf (24 hpi)

We selected in AphanoDB v2.0, the AeSSP cluster from scaffold2_1449549_1653277 (Fig. 6b) and scaffold2_134062_1449527 to evaluate the effector function of AeSSP genes. These clusters are unique, since in addition to their expression at the beginning of the interaction, they contain AeSSPs with a predicted NLS (AeSSP1251 and AeSSP1254, AeSSP1256), suggesting that these proteins could be translocated to the host cell to target nuclear components. To investigate if the AeSSPs can play a role in virulence, *Nicotiana benthamiana* leaves transiently expressing the AeSSPs were infected with *P. capsici* (Fig. 7a). In the assay, one side of the leaf was infiltrated with *Agrobacterium tumefaciens* containing an AeSSP:green fluorescent protein (GFP) fusion construct (AeSSP1250, AeSSP1254, AeSSP1255, and AeSSP1256), while on the other side of the leaf GFP was delivered alone. Quantification of the lesion surfaces at 3 dpi showed that AeSSP1256 significantly enhanced the susceptibility of *Nicotiana* compared to GFP alone, while no differences were observed for AeSSP1250, AeSSP1254, and AeSSP1255 (Fig. 7b). This indicates that AeSSP1256 may efficiently contribute to oomycete pathogenicity.

To investigate the subcellular localization of AeSSPs, chimeric genes encoding AeSSPs independently with (full-length version) or without (matured form) their own signal peptide under the control of the *CaMV35S* promoter were generated in fusion with a GFP marker. As shown in Fig. 7c, both versions of AeSSP1250 are detected either in the nucleus or the cytoplasm of *Nicotiana* cells, while AeSSP1255 versions are mainly cytoplasmic. The NLS-containing AeSSP1254 and AeSSP1256 are nuclear localized without any labeling of the nucleolus. The pattern of fluorescence is similar with the full version or the matured form of AeSSP1254 and AeSSP1256. We noticed that the expression of both versions of AeSSP1256 led to the detection of fluorescence as a ring surrounding the nucleolus in addition to filament-like structures. A closer view of AeSSP1256 in fusion with its own signal peptide confirmed the accumulation around the nucleolus and also as filament-like structures (Fig. 7d). This unexpected nuclear localization is reminiscent of the one observed with the cytoplasmic effector CRN79_188 from *Phytophthora capsici* upon its transient expression in *Nicotiana* leaf [63].

**Fig. 8:**
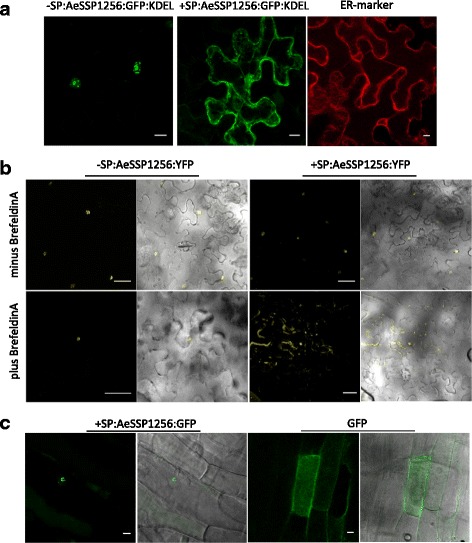
AeSSP1256 is a secreted protein which acts within the plant cell. **a** Overview of transiently transformed leaf epidermis with *A. tumefaciens* carrying constructs encoding for a matured (*–SP:AeSSP1256:GFP:KDEL*) or a full-length (*+SP:AeSSP1256:GFP:KDEL*) form of AeSSP1256 in fusion with an ER retention signal. Control leaves are transformed with an ER-marker. Confocal imaging was performed 24 hpi. Scale bar = 10 μm. **b** *Nicotiana* leaves expressing either the matured form (*–SP:AeSSP1256:YFP*) or the full-length form of AeSSP1256:YFP (*+SP:AeSSP1256:YFP*) before (*top panel*) and after (*above panel*) treatment with Brefeldin A, a drug that blocks transport of secretory proteins to the Golgi apparatus. Confocal imaging was performed 48 h post agroinfection. Scale bar = 45 μm. **c** Composite *Medicago* roots transformed with +SP:AeSSP1256:GFP or a GFP empty vector. Photographs are taken 15 days after *A. rhizogenes* inoculation

Since in all cases the presence of the native signal peptide did not alter the localization of the corresponding AeSSP, we tested whether the AeSSP entered the plant secretory pathway. A functional endoplasmic reticulum (ER) retention signal (KDEL motif) was added at the C-terminal of the full-length form of AeSSP1256 to trap the protein in the ER. In *Nicotiana* leaves expressing a +SP:AeSSP1256:GFP:KDEL construct, an extensive network throughout the cytoplasm was labeled as observed with an ER-marker [64] (Fig. 8a). In contrast, expression of the matured form –SP:AeSSP1256:GFP:KDEL produced fluorescence in the nucleus. This indicates that +SP:AeSSP1256 is delivered to the ER secretion pathway and that the predicted signal peptide of AeSSP1256 is active in *N. benthamiana*. To confirm AeSSP1256 trafficking in *Nicotiana* cells, we used the Brefeldin A (BFA) drug. BFA inhibits transport from the ER to the Golgi and causes the formation of membranous islands throughout the cell [65]. As expected, the localization of the full-length version of AeSSP1256 is disrupted by BFA, while the treatment did not affect the localization of the matured form. Thus, only +SP:AeSSP1256 transits through the Golgi in *Nicotiana* (Fig. 8b). Finally, we checked the effect and subcellular localization of AeSSP1256 in *Medicago* composite plants expressing the +SP:AeSSP1256:GFP construct. The expression of AeSSP1256 did not provoke any necrosis or alteration of root system development (data not shown). As illustrated in Fig. 8c, AeSSP1256 in fusion with its own signal peptide is nuclear localized 15 days after root transformation by *A. rhizogenes*, as observed previously in *Nicotiana* leaf.

## Discussion

In this study, we used a combination of genomics and transcriptomics approaches to identify gene repertoires involved in the adaptation of *Aphanomyces* pathogens to plant or animal hosts. This work provides the first genomics insight into the *Aphanomyces* genus, allowing one to precisely locate the phylogenetic position of *Aphanomyces* spp*.* within the oomycete lineage and providing clues for understanding animal and plant pathogenic evolution among oomycetes. Phylogenetic analysis suggested that specialization of *Aphanomyces* species to plant or animal hosts is an ancient event (more than 50 Mya), and analysis of gene contents revealed that phytopathogenic *A. euteiches* possesses a large and diverse repertoire of genes coding cell wall-degrading enzymes (CWDEs) which target plant cell wall polysaccharides, absent in *A. astaci*. In turn, *A. astaci* shows an expansion of protease genes, and during evolution it acquired genes coding proteins predicted to target chitin, the main component of the crayfish shell. These results indicate that host specialization is correlated with the presence of secretomes, which has been shaped by various evolutionary events including gene acquisition, gene losses, and gene amplification. Interestingly, transcriptome analyses showed that most of the genes coding enzymes able to degrade plant cell wall polysaccharides such as pectins and hemicellulases are strongly expressed during pathogenesis, strengthening their role in pathogenesis. Analyses performed on distantly related oomycete species, notably *Phytophthora* and *Saprolegnia*, gave similar results [31, 43, 66, 67], pointing out the key roles of degrading enzymes for pathogenesis and adaptation to specific ecological niches.

These results also raised the question of the origin of CWDE sequences in the *Aphanomyces* genus. Most of the secreted *A. euteiches* CWDEs acting against plant wall polysaccharides, such as pectinases and hemicellulases, show strong similarities to *Phytophthora* enzymes. Some of these genes have been suggested to be acquired by lateral gene transfer from a fungal donor, notably in *Phytophthora* [47, 48]. A striking example is a pectate lyase gene [48]. Interestingly, several paralogs of this gene were found in *A. euteiches* but not in *A.astaci,* suggesting that this gene was acquired before the divergence between the Saprolegnian and Peronosporalean lineages and was lost by *A. astaci* during adaptation to animal hosts. Availability of more oomycete genomes is needed to further define the origin of oomycete CWDEs involved in interactions with plants; however, our results indicate that acquisitions of plant CWDEs in oomycetes occur early during oomycete evolution.

By combining comparative genomics and transcriptomics, we identified a large set of SSPs which may represent new oomycete putative effectors. Among the 296 AeSSP genes, 120 are induced during at least one infection condition analyzed. Functional studies on four of these candidates revealed that these proteins are localized in various subcellular compartments, and one of them enhanced plant susceptibility to oomycete infection. Large repertoires of SSPs have been evidenced upon genome annotation of fungi interacting with plants [68, 69], animals [70], and insects [71]. Also unexpected was the large repertoire of SSPs predicted in mycorrhizal fungi [72, 73]*.* SSPs were also recently reported in bacteria such as the plant pathogen *Pseudomonas syringae* [74], but up to now no SSPs were described in oomycete genomes. Comparative fungal genomics studies showed evidences of rapid evolution of SSPs in related pathogens with different host ranges [70, 75]. A survey on 136 fungal secretomes archived in the Fungal Secretome Database (FSD) established that microorganisms living in close interaction with their hosts (symbiotic organisms, biotrophs) have commonly higher proportions of species-specific SSPs than necrotrophs or hemibiotrophs [76]. SSPs are frequently lineage specific and associated with host adaptation/specialization and are considered as putative effectors. Therefore, our work makes SSPs new candidates to be crucial players in oomycete adaptation to new hosts, particularly in species lacking the large family of RxLR effectors found in the Peronosporalean lineage.

## Conclusion

In this paper, we have analyzed the genomes of two close *Aphanomyces* species with the aim of identifying genetic determinants involved in host adaptation. However, the divergence between *A. euteiches* and *A. astaci* is still substantial, and these two species occupy two distinct ecological niches (soil *vs* aquatic environments). Thus, it is expected that many of the differences observed do not apply exclusively to pathogenesis but also to adaptation to these ecological niches. Importantly, it has been suggested that effectors can also play a role in competition or co-operation with other microorganisms occupying the same ecological niche. Sequencing of more *Aphanomyces* species is underway and will certainly help to refine the set of genes involved in host pathogenesis.

## Methods

### *Aphanomyces* spp. isolates and DNA preparation

The mycelia of *A. euteiches* isolate ATCC201684, *A. astaci* (Genotype E, strain Li07, provided by A. Petrusek, Czech Republic), and *A. stellatus* isolate CBS 578.67 were grown for 4 days in liquid YG medium (2.5% yeast extract, 5% glucose) at 23 °C in the dark. Biological samples were frozen in liquid nitrogen and the DNA extracted as reported in [31].

### *Aphanomyces euteiches* genome sequencing and assembly

CNS-Genoscope, Evry, France performed the sequencing and assemblies of the *A. euteiches* ATCC201684 genome, using a combination of Sanger and Illumina technologies. For 454 libraries of *A. euteiches*, DNA was extracted and fragmented to a range of 5–10 kb or around 20 kb using a HydroShear instrument. Fragments were end-repaired, and the extremities were ligated to 454 circularization adapters. Fragments were size selected respectively to 8 kb or 20 kb through regular gel electrophoresis and circularized using Cre-Lox recombination. Circular DNA was fragmented again by nebulization or using the Covaris E210 instrument (Covaris, Inc., Woburn, MA, USA). Fragments were end-repaired and ligated with library adapters. Mate-pair libraries were amplified and purified. Single-stranded libraries were isolated, then bound to capture beads and amplified in an oil emulsion (emPCR). The libraries were then loaded on a picotiter plate and sequenced using a GS FLX sequencer according to the manufacturer’s protocol. We also prepared 454 single-end read libraries according to the Roche standard procedure using RL (GS FLX Titanium Rapid Library Preparation Kit, Roche Diagnostics, Indianapolis, IN, USA). The libraries were sequenced using a 1/4 Pico Titer Plate on a 454 GS FLX instrument with Titanium chemistry (Roche Diagnostics). For the Illumina library, 2 μg of genomic DNA (gDNA) of *A. euteiches* was sheared to a 150–700-bp range using the Covaris E210 instrument (Covaris, Inc.). Sheared DNA was used for Illumina library preparation according to a semiautomatized protocol. Briefly, end repair, A tailing, and Illumina-compatible adapter (BiooScientific) ligation were performed using the SPRIworks Library Preparation System and SPRI TE instrument (Beckman Coulter, Simsbury, CT, USA) according to the manufacturer’s protocol. A 300–600-bp size selection was applied in order to recover most of the fragments. The DNA fragments were amplified by 10 cycles of PCR using Kapa HiFi HotStart DNA Polymerase (Life Technologies) and Illumina adapter-specific primers. The libraries were purified with 0.8× AMPure XP beads (Beckman Coulter). After library profile analysis with an Agilent 2100 Bioanalyzer (Agilent Technologies, Santa Clara, CA, USA) and qPCR quantification, the library was sequenced using 100 base-length read chemistry in paired-end mode on the Illumina HiSeq2000 (Illumina, Canton, MA, USA). All reads were assembled with Newbler version vMapAsmResearch-04/19/2010-patch-08/17/2010. The 6319 contigs were linked into 349 scaffolds. The sequence quality of scaffolds from the Newbler assembly was improved as described previously [77] by automatic error corrections with Solexa/Illumina reads (146-fold genome coverage), which have a different bias in error type compared with 454 reads. Finally, the assembly was gap closed using Illumina data and GapCloser [78]. Putative misassemblies were identified and corrected using default parameters of REAPR (version 1.0.17) [79]. Assembly completeness was estimated using BUSCO v3 [35] based on a set of common fungal genes (F) or Alveolata/Stramenopiles genes (AS), aka benchmarking universal single-copy orthologs (BUSCOs). We found 83.1% (F)/78.6% (AS) complete BUSCOs, 3.8% (F)/0% (AS) duplicated BUSCOs, and 3.1% (F)/1.7% (AS) fragmented BUSCOs, leading to 13.8% (F)/19.7% (AS) missing BUSCOs in *A. euteiches*. Statistics for different sequencing technologies performed in this study for the *A. euteiches* ATCC201684 genome are presented below.

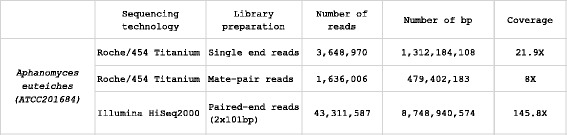



### *Aphanomyces astaci* and *Aphanomyces stellatus* genome sequencing and assemblies

The GeT-PlaGe core facility, Toulouse, France realized Illumina sequencing of the *A. astaci* and *A. stellatus* genomes*,* and the assemblies were performed by the Genotoul Bioinformatic Platform, Toulouse, France. DNA-seq libraries were prepared using an Illumina TruSeq DNA v2 Library Prep Kit following the manufacturer’s instructions. DNA was fragmented by sonication, size selection was performed using E-Gel 0.8% (Thermo Fisher Scientific, Waltham, MA, USA), and the adapters were ligated. Ten PCR cycles were applied to amplify the library before final purification with Agencourt AMPure XP beads (Beckman Coulter). Library quality was assessed using an Agilent Bioanalyzer, and the libraries were quantified by qPCR using the Kapa Library Quantification Kit. DNA-seq experiments were performed on an Illumina HiSeq2000 Sequencer using a paired-end read length of 2 × 100 pb with the HiSeq v3 Reagent Kit. The raw reads have been quality checked and stored in NG6 [80]. FastQC (version 0.10.0) (http://www.bioinformatics.babraham.ac.uk/projects/fastqc/) was used to produce quality metrics and bwa aln (version 0.6.1-r104) to search *Escherichia coli*-, yeast-, and phage-contaminated reads. The reads of *A. astaci* and *A. stellatus* were assembled using MaSuRCa, version 2.0 [81], and the assembly metrics were calculated using the assemblathon_stats.pl script [82].

### Structural and functional annotation of *Aphanomyces* genomes

The assembled data of *Aphanomyces* were annotated with Augustus v2.757 [83] trained with the assembled RNA-seq transcript generated in this study and the publicly available ESTs previously obtained from *A. euteiches* [28] using autoAug.pl [84] and PASA [85]. RNA-seq reads were first assembled with trinityrnaseq_r2012–10-05 [86]. The accuracy of the prediction was evaluated by mapping the RNA-seq reads to the genomes using bwa [87]. Genes were annotated using BLASTP against the RefSeq database [88]. For protein family classification, InterProScan [89] and the Pfam protein domain database [90] were used. Gene ontologies were classified based on InterProScan annotation IDs. Kyoto Encyclopedia of Genes and Genomes (KEGG) and Enzyme Commission (EC number) data were obtained with KEGG Automatic Annotation Server (KAAS) predicted protein sequences [91] and were also mapped to the Clusters of Orthologous Groups (COG) classification system [92]. Predictions of signal peptides were performed using SignalP 4.1 [93]. Carbohydrate-Active enZymes (CAZymes) in the protein models were predicted using the dbCAN pipeline [94]. Orthology comparisons between the predicted *Aphanomyces* genomes and protein datasets from nine deeply sequenced oomycete genomes were performed via OrthoMCLcompanion [95] using standard parameters. Interspersed repeat sequences were de novo identified in the genomes of *A. euteiches, A. stellatus*, and *A. astaci* using the RepeatModeler 1.0.8 pipeline [96]. Short (< 1000 bp) Class II transposons that were devoid of recognizable open reading frames were classified as miniature inverted-repeat transposable elements (MITEs). When possible, we assigned them to a superfamily based on the nature of their target site duplication (TSD): piggybac (TTAA motif), Tc1-mariner (TA motif), and PIF-Harbinger (TTA or TAA motif) [97]. The remaining RepeatModeler unclassified consensus sequences (producing no BLASTX hits and having no distinct boundaries) were classified as “unclear”. A library of repeated sequences was then constructed for each species and used to mask each genome with RepeatMasker 4.0.5 [96]. To characterize the global evolutionary dynamics of *Aphanomyces* TEs, we plotted the frequency distribution of the percent divergence between each consensus and all their cognate copies. To assess the extent to which the TEs are related to each other and to other known TEs, we used each library as a query to perform BLASTN searches on the other libraries and on the Repbase [98] library of TEs (e-value cut-off = 10–20). Gene annotations were visualized in Apollo [99]. All the data generated in this work were incorporated in an updated version (version 2.0) of AphanoDB [37, 100].

### Preparation of RNA material

Seeds of *M. truncatula* Gaertn. F83005.5 were scarified, sterilized, and in vitro cultured as previously described [101, 102]. Roots of 10-day-old plants were inoculated with *A. euteiches* ATCC201684 zoospores as described in [103]. Infected roots were harvested 1 dpi, 3 dpi, and 9 dpi. In parallel, flasks with 50 mL PG medium (20.0 g/L animal peptone; 5.0 g/L glucose) were inoculated with 1000 zoospores of *A. euteiches*. Mycelium samples were collected from the PG medium 1 dpi, 3 dpi, and 9 dpi. Zoospores were collected by centrifugation at 14.000× g at 4 °C for 45 min. For all samples three biological replicates were collected. RNA was isolated using the RNA Plant Mini Kit (QIAGEN, Hilden, Germany) according to the manufacturer’s protocol, except for the DNAse treatment (RNase-free DNase, QIAGEN), which was done on the column for 20 min. The RNA concentration was determined, and the quality of the RNA was verified using a Fragment Analyzer (Advanced Analytical, Ankeny, IA, USA).

### RNA sequencing

Illumina sequencing of RNA samples generated from *A. euteiches* mycelium (MY) and mycelium grown in contact with *M. truncatula* roots (INT) [28], was performed by CNS-Genoscope, Evry, France. Starting with 2 μg of total RNA, double-stranded cDNA was first generated using the TruSeq RNA sample prep kit (Illumina, Canton, MA, USA), and then paired-end libraries were prepared using NEBNext Sample Reagent Set (New England Biolabs, Ipswich, MA, USA). Briefly, the messenger RNAs (mRNAs) were polyA-selected, chemically fragmented, and converted into single-stranded cDNA using random hexamer priming. The second strand was generated to create double-stranded cDNA. The cDNA was then end-repaired and 3’-adenylated, and Illumina adapters were added. The ligation products were purified and the DNA fragments (> 200 pb) were PCR-amplified using Platinum Pfx DNA Polymerase (Life Technologies) and Illumina adapter-specific primers. After library profile analysis by the Agilent 2100 Bioanalyzer (Agilent Technologies) and qPCR quantification (MxPro, Agilent Technologies), the libraries were sequenced on an Illumina HiSeq2000 instrument using 101 base-length read chemistry in a paired-end mode. Statistics for INT and MY libraries are reported below.

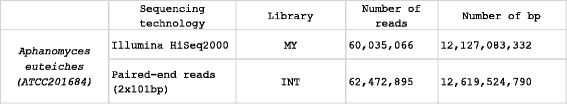



In this study new RNA samples were generated from *A. euteiches* mycelium, zoospores, or infected *M. truncatula* roots (1 dpi, 3 dpi, and 9 dpi) according to [103, 104]. RNA-seq libraries were prepared according to Illumina’s protocols using the Illumina TruSeq Stranded mRNA Sample Prep Kit to analyze mRNA, at the GeT-PlaGe core facility, Toulouse. Briefly, mRNA was selected using poly-T beads, and cDNA was generated using random hexamer priming, and adapters were then added. Ten cycles of PCR were applied to amplify the libraries. Library quality was assessed using an Agilent Bioanalyzer, and the libraries were quantified by qPCR using the Kapa Library Quantification Kit. RNA-seq experiments have been performed on an Illumina HiSeq2500 using a paired-end read length of 2 × 100 pb with the Illumina TruSeq SBS Sequencing Kits v3. Statistics are presented in the manuscript.

### RNA-sequencing data analysis

Reads obtained from the different life stages of *A. euteiches* (zoospore, mycelium) or infected roots (1 dpi, 3 dpi, and 9 dpi), were mapped to the *A. euteiches* genome with CLC Genomics Workbench 10.1.1 (QIAGEN). Predefined settings were used except for the similarity fraction, which was set at 0.98, and the read counts per gene (paired reads count as one) were exported. Using the R package edgeR [105], the data were filtered for genes with low counts across all libraries (counts per million (cpm) > 2 in at least three libraries) and then trimmed mean of M (TMM) normalized [106], the tagwise dispersion was estimated, and the differential expression was calculated. Using the generalized linear model likelihood ratio test, *p* values were obtained, and multiplicity correction was performed by applying the Benjamini-Hochberg method [107, 108]. The multi-dimensional scaling plot was created using the 500 genes with the highest dispersion over all libraries. The enrichment of GO terms in up- or downregulated genes was tested using a Fisher’s exact test with the classical algorithm in the topGO R package [109]. Heatmaps were created using the gplots R package [110], and clustering was performed according to the complete linkage method with Euclidean distance measure.

### Phylogenetic and divergence time analysis of *Aphanomyces* species

To assess the phylogenetic position and divergence times of *A. astaci*, *A. euteiches*, and *A. stellatus* within oomycetes, we followed the methodology of Matari and Blair [38], who produced a robust timetree of oomycetes. We used the amino acid alignments of 40 genes that were assembled for 17 oomycetes and one outgroup species (*Tetrahymena thermophila*), to which we added the sequences of the three *Aphanomyces* species. These 40 datasets were selected among 70 initial alignments of genes involved in regulation of gene expression, because there was strong evidence supporting their orthology relationships and they contained minimal missing data [38]. Amino acid sequences were retrieved from the proteomes of the three *Aphanomyces* species using sequences of the three Saprolegniales (*Thraustotheca clavata*, *Saprolegnia parasitica*, and *Achlya hypogyna*) as queries in BLASTP searches. Most genes could be identified for the three *Aphanomyces* species, except six in *A. astaci* (*HMG-CBF-BFY*, *MnmA*, *p15*, *Ssl1*, *TAF6*, *TAF12*) and one in *A. stellatus* (*TFIIB*). The 40 alignments were then concatenated and subjected to a maximum likelihood phylogenetic analysis in PhyML v3.0 [111] using the WAG model of amino acid substitutions as in [38]. The robustness of the tree was evaluated by performing 1000 bootstrap replicates. Divergence times were conducted in BEAST v1.8.2 [112] using the same parameters as in [38]. Briefly, we performed 50 million generations run using the PhyML-generated tree as a guide tree and treating each of the 40 datasets as a separate partition with the WAG substitution model, a Yule speciation process (uniform distribution; 0–100; initial value 0.01), and the random local clock model, which was shown to best fit the data by [38]. The calibration strategy follows that of [38] and consists of three calibration points modeled with a gamma prior distribution: (1) diatom node: 5–95% quantiles = 74–100 Myrs, (2) diatom + *Ectocarpus* node: 5–95% quantiles = 176–202 Myrs, and (3) oomycetes + ochrophytes node: (5–95% quantiles = 418–550 Myrs). The root age was modeled using a uniform prior distribution (408–1750 Myrs; initial value 635). We used Tracer v1.6 [113] to visualize convergence and determine the burn-in and FigTree v1.4 [113] to generate the dated tree of oomycetes.

### Construction of plasmid vectors and *Agrobacterium-*mediated transformations

The AeSSPs sequences were amplified by PCR from *A. euteiches* gDNA with specific primers (Additional file 6: ST3b). The CACC cloning site was added to each forward primer. PCR products were purified using the PCR Clean-Up Kit (Promega, Madison, WI, USA) and introduced in the pENTRY-D-TOPO vector (pENTR/D-TOPO Cloning Kit, Invitrogen). Positive clones were introduced in a pK7FWG2 vector (Invitrogen) or pAMpAT/YFP vector. After sequencing, positive clones were introduced in *A. tumefaciens* GV3101 and *A. rhizogenes* ARqua1. For the KDEL fusion, the GFP construct was amplified from the obtained pK7FWG2 vector by PCR using primers that introduced a C-terminal SEKDEL sequence (Additional file 6: ST3b). Cloning was performed as previously reported using a pK2GW7 vector (Invitrogen). For leaf infiltration, *A. tumefaciens* GV3101-transformed strains were syringe-infiltrated as described in [61]. For *M. truncatula* roots transformation, *A. rhizogenes* ARqua1 strains were used and confocal imaging was performed at 28 dpi, as described in [29].

### Brefeldin A treatment and *P. capsici* infection assay

Brefeldin A (BFA, Sigma-Aldrich, St. Louis, MO, USA) treatment was performed 24 h after agroinfiltration of *N. benthamiana.* Leaf discs were vacuum infiltrated with TBS1X (50 mM Tris-HCl; 150 mM NaCl pH 7.6) and 15 μg/mL BFA for 30 min, and incubated for 24 h at room temperature. Leaf discs were washed in TBS1X before confocal imaging. For the infection assay, *Phytophthora capsici* LT3112 was grown on V8 agar plates for 7 days at 22 °C. Zoospore preparation and inoculation on agroinfiltrated *N. benthamiana* leaf and symptom measurement were performed as reported in [29].

### Confocal microscopy

Imaging was performed on Leica DM6B-Z-CS or Leica AOBS TCS SP2 SE laser scanning confocal microscopes. Excitation wavelengths and emission filters were 488 (GFP) or 514 nm (YFP) long-pass. Images were acquired with a 40× water immersion lens or a 25× Fluotar Visir water objective, and corresponded to Z projections of scanned tissues. Image processing was performed using ImageJ software including three-dimensional reconstruction to compute projections of serial confocal sections.

## Additional files

Additional file 1: **ST1.** Genome features and annotations. 1a. Genome resources and nomenclature used in this study. 1b. BUSCO analysis. 1c. TE analysis. 1d. Orthology analysis (OrthoMCL) summary (nine oomycetes and *Ae, Aa*). 1e. OrthoMCL, group ID (11 oomycete proteomes). 1f*. Ae, Aa*: predicted secreted genes ID. 1g. *Ae, Aa*: GO analysis (secretome). 1h. *Ae, Aa*: specific secretomes analysis. 1i. CAZyome analysis. 1j. Fungal and oomycete pectate lyase sequences. 1k. CRN effectors predicted in *Ae* and *Aa* genomes. (XLSX 2962 kb)

Additional file 2: **Figure S1.** Phylogenetic analysis of the *A. euteiches* PL1 gene family. A maximum likelihood phylogeny analysis was performed on an oomycete and fungal PL1 sequence alignment using the neighbor joining construction methods and the WAG protein substitution model. Bootstrap analysis was performed with 1000 replicates. (PPTX 89 kb)

Additional file 3: **ST2.** *A. euteiches* expression analysis. 2a. Statistics of RNA-seq experiments. 2b. Percentage of up- and downregulated *A. euteiches* genes. 2c. Overrepresented GO terms in zoospores vs mycelium. 2d. Overrepresented GO terms at 3 dpi vs mycelium. 2e. Overrepresented GO terms at 9 dpi vs mycelium. 2f. Expression data for heatmap construction (GH). 2g. Expression data for heatmap construction (PL). 2h. Expression data for heatmap construction (proteases). 2i. Expression data for heatmap construction (CE). 2j. Expression data for heatmap construction (CBM). 2k. Expression data for heatmap construction (protease inhibitor). (XLSX 131 kb)

Additional file 4: **Figure S2.** RNA-seq samples relationship. Multi-dimensional scaling plot (Euclidean distance; top = 500 genes) showing the leading log2-fold change (leading logFC) between the normalized samples of *A. euteiches.* Three biological replicates per condition. (PPTX 85 kb)

Additional file 5: **Figure S3.** Gene expression of *A. euteiches*. Heatmaps of the Log2 RPKM values of carbohydrate esterases (A), carbohydrate-binding module (B) and proteases inhibitors (C). *Colors on the left* of the heatmaps indicate the subgroup the CE, the CBM or the PInh belongs to and if present the group of the corresponding class, and *colors on the right* indicate if the genes are significantly up- (*red*) or down- (*blue*) regulated in zoospores, infected roots 3 dpi and infected roots 9 dpi compared to expression in mycelium grown in vitro. (PPTX 99 kb)

Additional file 6: **ST3.** *A. euteiches* small secreted proteins (AeSSPs). 3a. AeSSP classification and expression. 3b. Primers used in this study. (XLSX 37 kb)

## Abbreviations

BFA: Brefeldin A
CBM: Carbohydrate-binding modules
CRN: Crinkler
CWDE: Cell wall-degrading enzyme
GH: Glycosyl hydrolase
GO: Gene Ontology
PL: Polysaccharide lyase
SSP: Small Secreted Protein
TE: Transposable Element
