# Supplementary figures and images for: Genomics analysis of Aphanomyces spp. identifies a new class of oomycete effector associated with host adaptation
Source: BMC Biol. 2018 Apr 18;16:43. doi: 10.1186/s12915-018-0508-5 (PMC5907361; doi:10.1186/s12915-018-0508-5)

## Slide 1
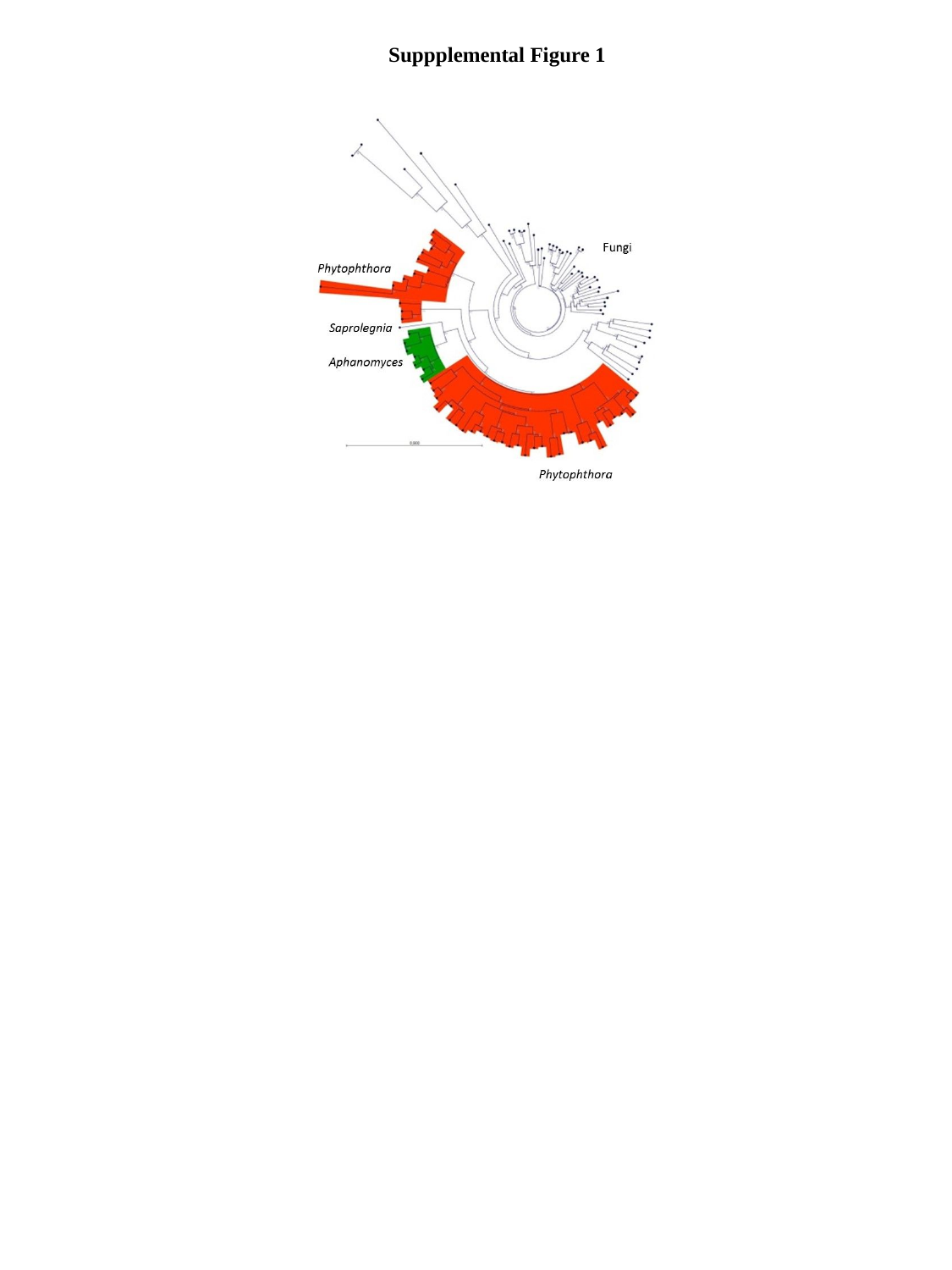

Suppplemental Figure 1

Supplement: Supplementary file 2 — Figure S1. Phylogenetic analysis of the A. euteiches PL1 gene family. A maximum likelihood phylogeny analysis was performed on an oomycete and fungal PL1 sequence alignment using the neighbor joining construction methods and the WAG protein substitution model. Bootstrap analysis was performed with 1000 replicates. (PPTX 89 kb) [file 12915_2018_508_MOESM2_ESM.pptx]

## Slide 1
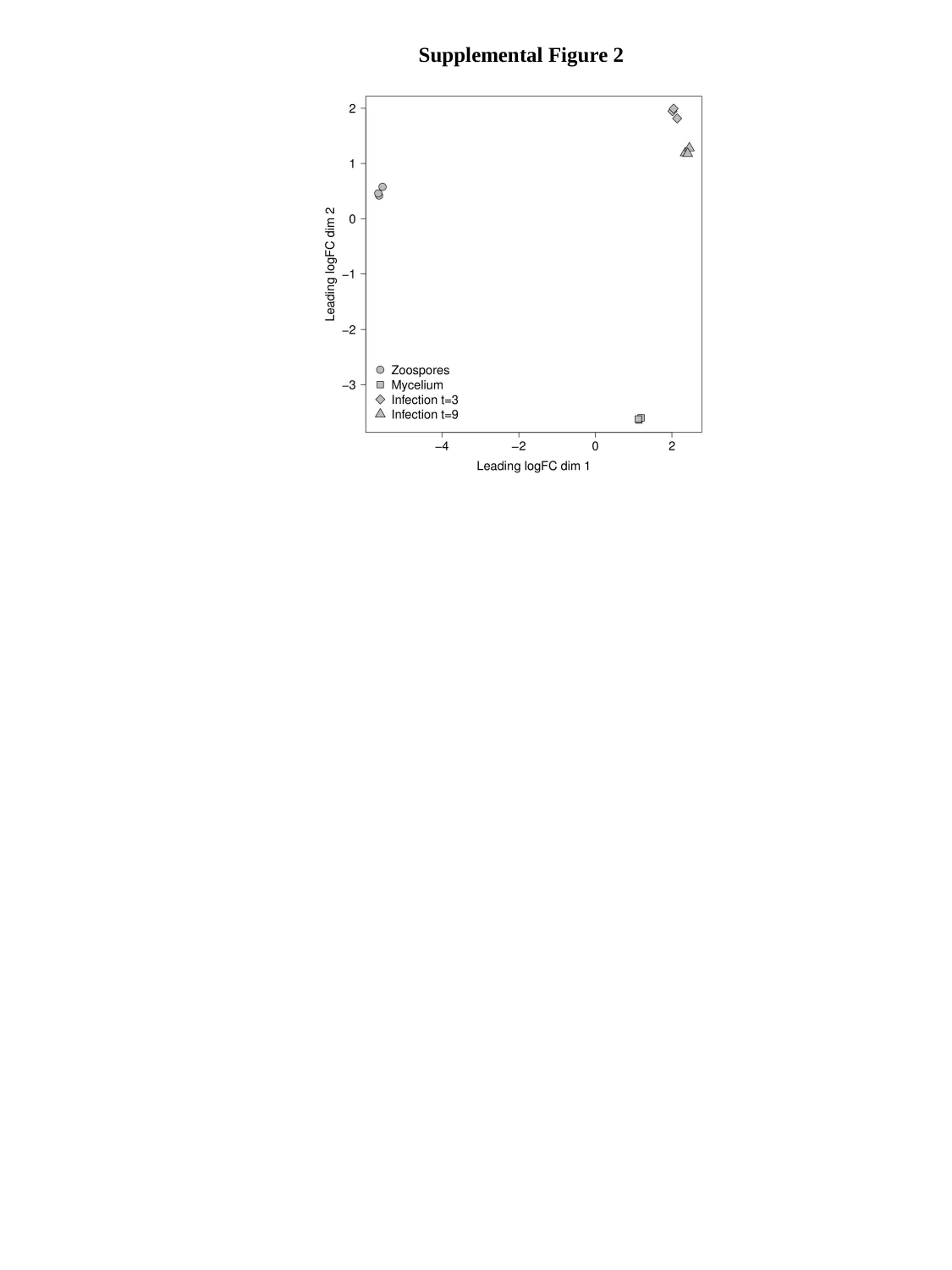

Supplemental Figure 2

Supplement: Supplementary file 4 — Figure S2. RNA-seq samples relationship. Multi-dimensional scaling plot (Euclidean distance; top = 500 genes) showing the leading log2-fold change (leading logFC) between the normalized samples of A. euteiches. Three biological replicates per condition. (PPTX 85 kb) [file 12915_2018_508_MOESM4_ESM.pptx]

## Slide 1
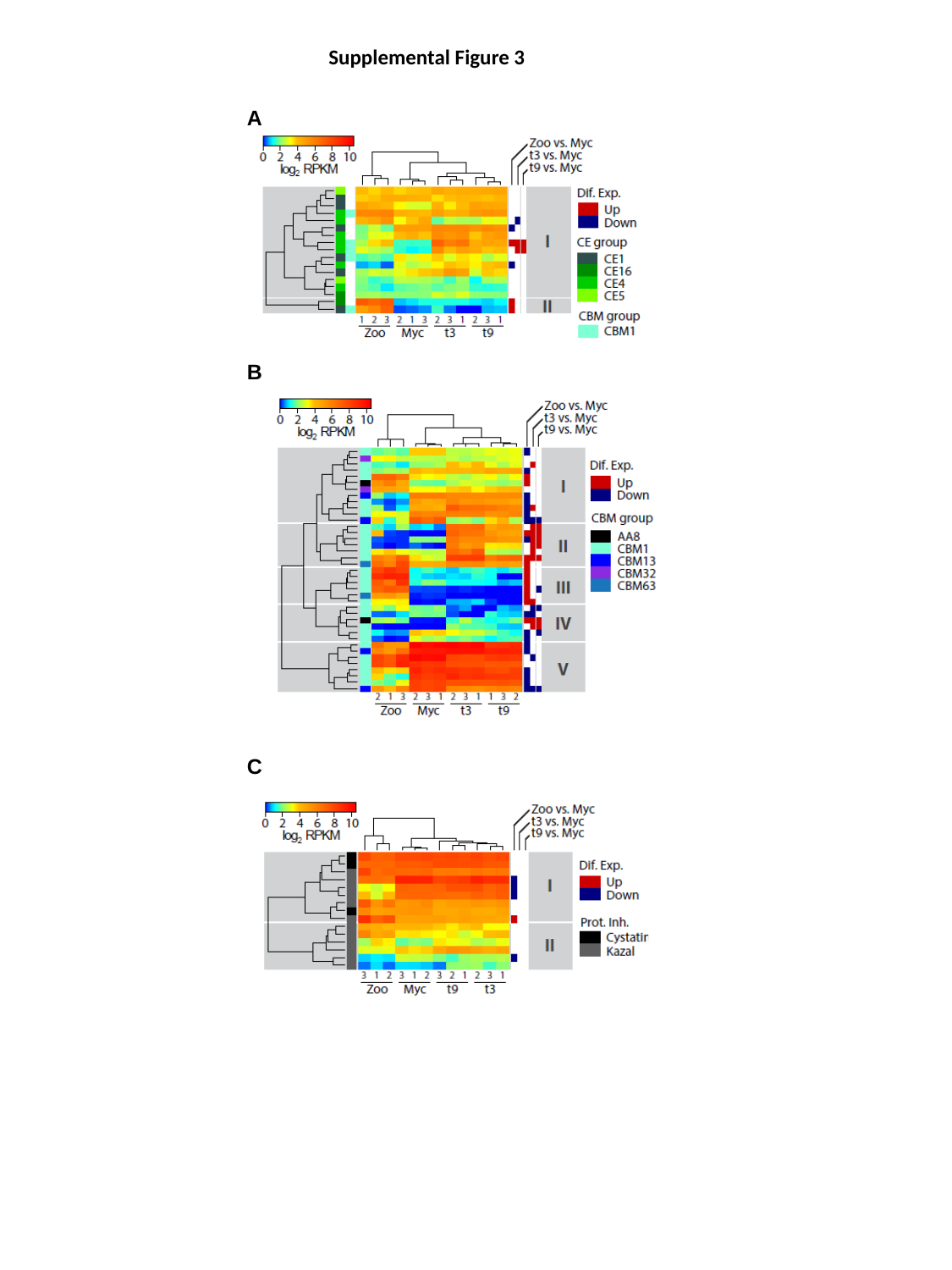

Supplemental Figure 3
A
B
C

Supplement: Supplementary file 5 — Figure S3. Gene expression of A. euteiches. Heatmaps of the Log2 RPKM values of carbohydrate esterases (A), carbohydrate-binding module (B) and proteases inhibitors (C). Colors on the left of the heatmaps indicate the subgroup the CE, the CBM or the PInh belongs to and if present the group of the corresponding class, and colors on the right indicate if the genes are significantly up- (red) or down- (blue) regulated in zoospores, infected roots 3 dpi and infected roots 9 dpi compared to expression in mycelium grown in vitro. (PPTX 99 kb) [file 12915_2018_508_MOESM5_ESM.pptx]
